# Prenatal and Postnatal Polycyclic Aromatic Hydrocarbon Exposure, Airway Hyperreactivity, and Beta-2 Adrenergic Receptor Function in Sensitized Mouse Offspring

**DOI:** 10.1155/2013/603581

**Published:** 2013-12-12

**Authors:** Sophie Chu, Hanjie Zhang, Christina Maher, Jacob D. McDonald, Xiang Zhang, Shuk-Mei Ho, Beizhan Yan, Steven Chillrud, Frederica Perera, Phillip Factor, Rachel L. Miller

**Affiliations:** ^1^Division of Pulmonary, Allergy, and Critical Care Medicine, Department of Medicine, Columbia University College of Physicians and Surgeons, PH8E-101B, 630 W. 168th Street, New York, NY 10032, USA; ^2^Department of Toxicology, Lovelace Respiratory Research Institute, Albuquerque, NM 87101, USA; ^3^Department of Environmental Health Sciences, University of Cincinnati College of Medicine, Cincinnati, OH 45201, USA; ^4^Lamont-Doherty Earth Observatory, Columbia University, Palisades, NY 10964, USA; ^5^Department of Environmental Health, Columbia University Mailman School of Public Health, New York, NY 10032, USA; ^6^Department of Medicine, University of Arizona, Tucson, AZ, 85721, USA; ^7^Division of Pediatric Allergy and Immunology, Department of Pediatrics, Columbia University College of Physicians and Surgeons, New York, NY 10032, USA

## Abstract

Despite data associating exposure to traffic-related polycyclic aromatic hydrocarbons (PAH) in asthma, mechanistic support has been limited. We hypothesized that both prenatal and early postnatal exposure to PAH would increase airway hyperreactivity (AHR) and that the resulting AHR may be insensitive to treatment with a **β**_2_AR agonist drug, procaterol. Further, we hypothesized that these exposures would be associated with altered **β**_2_AR gene expression and DNA methylation in mouse lungs. Mice were exposed prenatally or postnatally to a nebulized PAH mixture versus negative control aerosol 5 days a week. Double knockout **β**_2_AR mice were exposed postnatally only. Prenatal exposure to PAH was associated with reduced **β**_2_AR gene expression among nonsensitized mice offspring, but not increases in DNA methylation or AHR. Postnatal exposure to PAH was borderline associated with increased AHR among sensitized wildtype, but not knockout mice. In the first study that delivers PAH aerosols to mice in a relatively physiological manner, small effects on AHR and **β**_2_AR gene expression, but not **β**_2_AR agonist drug activity, were observed. If confirmed, the results may suggest that exposure to PAH, common ambient urban pollutants, affects **β**_2_AR function, although the impact on the efficacy of **β**_2_AR agonist drugs used in treating asthma remains uncertain.

## 1. Introduction

Exposure to traffic-related air pollution has been associated with exacerbations of respiratory symptoms, decreased lung function, and the development of asthma [1–5]. Incomplete combustion of diesel exhaust particles emitted by motor vehicle engines produces a complex mixture of pollutants that includes significant concentrations of polycyclic aromatic hydrocarbons (PAH). Previously, our group at the Columbia Center for Children's Environmental Health (CCCEH) and others have shown that exposure to PAH was associated with asthma in children [1, 4, 6, 7]. Notably, the prenatal time window of exposure to PAH has been implicated in the development of childhood asthma, particularly in the presence of exposure to secondhand smoke [4, 8]. Also, repeated prenatal and early childhood exposure to pyrene, the predominant PAH in the NYC CCCEH local environment, has been associated with asthma regardless of exposure to secondhand smoke or seroatopy in the CCCEH cohort [1]. Despite the emergence of data associating exposure of PAH to asthma-related outcomes, mechanistic support has been limited.

Inhaled *β*_2_-adrenergic agonists are common treatments for reactive airway diseases and used for short-term and long-term alleviation of bronchoconstriction [9]. They are believed to function by binding to the active site of *β*_2_ARs on airway epithelial and smooth muscle cells, leading to bronchodilation via activation of adenylyl cyclase, generation of intracellular cAMP, and the associated signaling events [10]. Despite their wide usage, inhaled *β*_2_AR agonists have been associated with serious asthma-related complications. Specifically, the extended use of short-acting agonists has been associated with a loss of their protective bronchodilatory action and severe asthma exacerbations [11]. Certain populations, like children, African Americans, and those with particular *β*_2_AR genotypes (e.g., homozygous for arginine at *β*_2_AR-16 [Arg/Arg]), appear more susceptible to the morbidity and even mortality associated with long-term *β*_2_ agonist use [12–14]. Mechanisms proposed for this increase in morbidity and loss of efficacy have included suppression of symptoms associated with early but not more advanced inflammation and tolerance to the bronchodilatory activity, possibly via receptor desensitization [11].

More recent evidence suggests that *β*_2_AR function and signaling could be impaired by exposure to PAH from ambient urban air. Specifically, we produced a PAH mixture that mimicked the proportional distribution of individual PAH observed in NYC air and found that exposure to the PAH mixture reduced the expression and function of *β*_2_AR in primary human and mouse cell systems [15]. This observation provided the first evidence that environmentally relevant concentrations of PAH can impede *β*_2_AR-mediated airway relaxation. Another study indicated that the exposure of adipocytes to PAHs also impaired the function of *β*_2_AR, without reducing membrane-bound receptor numbers [16].

The primary objective of this study was to determine if exposure to traffic-related PAH alters airway *β*_2_AR function following in utero and early life exposures *in vivo*. We hypothesized that both prenatal and early postnatal exposure to PAH would increase AHR in ovalbumin-sensitized offspring mice and that this AHR may not improve following administration of a *β*_2_AR agonist drug. Further, we hypothesized that these exposures would be associated with altered *β*_2_AR gene expression and DNA methylation in mouse lungs. The key to our approach was the administration of an aerosol of PAH with levels that mimicked the proportional distribution of individual PAH observed in NYC air inhaled by pregnant women [8, 17] that was delivered in a manner more physiological than reported in previous murine studies [18].

## 2. Materials and Methods

### 2.1. Animals

Seven-week-old C57/Bl6 mice were obtained from Charles River. The mice were housed in a conventional animal facility in a private room under a 12 hour light/dark cycle with free access to food and water. Double knockout *β*_2_AR DKO *β*_2_ *AR* ^*tm1Bkk*^ *β*_2_ *AR* ^*tm1Bkk*^/J mice (http://jaxmice.jax.org/strain/003810.html) with complete absence of *β*_2_ *AR* function (null for adrenergic receptor 1 and 2 genes) were obtained from Jackson laboratory. Published data showed that these mice do not respond to administration of *β* agonist drugs [19]. Mice were mated at 8 weeks old. These mice were housed and fed ad lib breeder chow (Labdiet, St. Louis, MO) starting the first day of mating until postnatal day (PND) 28 when it was replaced with regular chow. The experimental protocol was approved by the IACUC.

### 2.2. PAH Exposure

The PAH mixture was produced by the Lovelace Respiratory Research Institute to replicate the proportional distribution of individual PAH that was measured among a cohort of pregnant women participating at the CCCEH birth cohort [8, 17] as shown in Table 1. The negative control aerosol solution consisted of 99.97% purified water, 0.02% Tween 80, and 0.01% Antifoam A (Sigma-Aldrich, St. Louis, MO). 100 uL of PAH solution was added to yield the final concentration of 7.29 ng/m^3^. The aerosols were tested for bacterial growth annually using aerobic and anaerobic cultures (Charles River, MA). Fresh solutions were prepared weekly.

**Figure 1:**
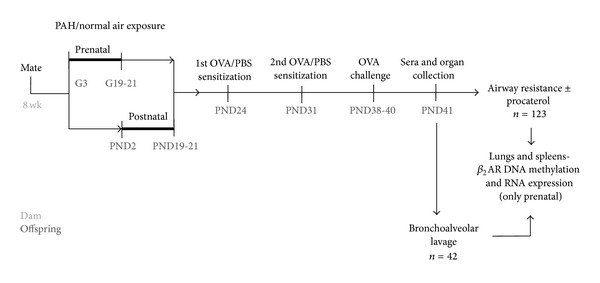
Algorithm of experimental design. Mice were exposed to PAH or normal air either on GD 3 to 19–21 or PND 2 to 19–21. Offspring were sensitized to either OVA or PBS on PND 24 and then again on PND 31. The mice were OVA challenged on PNDs 38–40. Airway resistance was measured and biospecimens were collected on PND 41.

Mice were exposed prenatally or postnatally to PAH versus negative control aerosol, as summarized in Figure 1. The 15 mL solutions were delivered via nebulizers (Unomedical Inc., McAllen, TX). Filtered compressed air was connected to the nebulizer. Prenatal aerosol was delivered beginning from gestational days (GD) 1–3 (the Monday after mating) until GD 19–21 or day of delivery. Postnatal aerosol was administered beginning from postnatal day (PND) 2 until PND 19–21.

**Figure 2:**
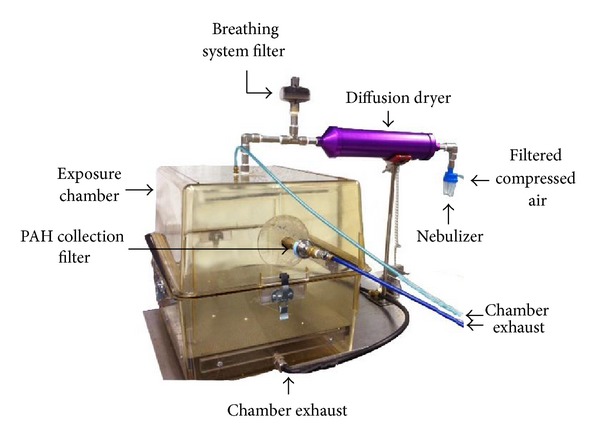
PAH chamber. Mice were exposed to PAH or normal air in two side-by-side chambers delivered via aerosol (only one shown). Filters were collected as depicted and used to measure ambient concentrations.

Two cages without filter tops were placed in each (PAH, normal air) exposure chamber for five hours a day, five days a week, as shown in Figure 2. The exposure chambers were set to achieve a flow of 12.5 to 13.0 liters per minute (LPM). Pressure gauges on the panel were set to 20 psi. At the beginning of each week of the exposure, a 25 mm Pall Flex filter (Pall Lifesciences, Port Washington, NY) with an Amberlite XAD-4 coating was combined with a noncoated filter in front to provide support to the brittle XAD coated filter. The filters were replenished weekly and stored in −20 F freezer until analyzed at Lamont-Doherty Earth Observatory, Columbia University. Three weekly filters with PAHs were extracted together by mixture of CH_2_Cl_2_ and CH_3_OH (9 : 1 v : v) 3 times under sonication and then concentrated under the gentle flow of N_2_. Levels of PAHs, including pyrene which was used as an indicator of PAH aerosolization, were measured by a Varian (now Agilent) 1200L gas chromatography-tandem mass spectrometer (GC/MS/MS).

### 2.3. Sensitization to Ovalbumin (OVA) and Measurement of OVA-IgE

Pups were sensitized to either ovalbumin (OVA) or phosphate buffered saline (PBS) as a negative control beginning from PND 24 using a 0.1 mg solution of Grade V chicken egg albumin (Sigma-Aldrich, St. Louis, MO, A5503-10G) in water and 4 mg Imject alum (Pierce, Rockford, IL, number 77161) intraperitoneal (i.p., 200 *μ*L). The mice were redosed one week later. All the mice were then challenged with aerosolized OVA (3% OVA in sterile PBS) for 30 minutes on days 38, 39, and 40.

Anti-OVA-immunoglobulin E (OVA-IgE) was measured by enzyme-linked immune sorbent assay (ELISA) in all available blood specimens using the Mouse Serum Anti-OVA IgE Antibody Assay Kit (Chondrex, Redmond, WA) according to the manufacturer's protocol. Sera were diluted from 1 : 5 to 1 : 10. The data were analyzed using a log-log model equation (http://readerfit.com/).

### 2.4. Measurement of AHR

Half of the mice were pretreated with 3 mM of the *β*_2_-adrenergic agonist procaterol (Sigma-Aldrich, St. Louis, MO) by aerosol for 30 minutes immediately prior to AHR measurement using a smaller chamber connected to a nebulizer. Airway reactivity was measured between PND 41 and 45 of age as shown in Figure 1. Airway resistance was measured using a computer controlled, small animal ventilator (SAV, SCIREQ flexiVent, Montreal, QC, Canada). Mice were sedated (pentobarbital, 30–40 mg/kg i.p.), tracheotomized with an 18 g × 1 cm metal cannula, paralyzed (pancuronium, 50 mg/kg i.p.), and ventilated using a quasisinusoidal ventilatory mode at 150 breaths per minute (bpm) with 3 cm H_2_O of positive end expiratory pressure. Body temperature was maintained with a water-perfused heating pad and cardiac rhythm was monitored continuously using a SCIREQ EKG. Lung volume was then standardized twice using a constant flow, pressure-limited mode of ventilation (tidal volume = 10 mL/kg, pressure at airway opening limited to 30 cm-H_2_O at 150 bpm), and occlusion of the expiratory port of the ventilator for 10 breaths. Mice were then allowed to equilibrate for 5 minutes during which they were ventilated with a quasisinusoidal pattern. Following equilibration, PBS was aerosolized using a SCIREQ zero-dead space ultrasonic nebulizer that was positioned in the inspiratory limb of the ventilator circuit. Airway resistance was measured using an 8-second forced oscillation, composed of nonoverlapping, nonharmonic frequencies that were used to produce an index of central airways resistance, R_*n*_ (Newtonian resistance). Incremental doses of methacholine (8, 16, 32, and 64 mg/mL in PBS) were then aerosolized. For each animal, peak airway resistance following each dose of methacholine was normalized to R_*n*_ following PBS and plotted. All measures were graded A, B, C, or D according to prespecified criteria: (A) an ideal curve exhibiting a strong initial peak of resistance and then a return to baseline following each dose of methacholine, (B) as in (A), but one methacholine doses without clearly defined peak, (C) as in (B), but more than methacholine doses without clearly defined peak, or many exclusions noted by the software, or (D) flat R_*n*_ or loss of cardiac rhythm during procedure. Only data graded A–C were used. At the end of the procedure, all mice were euthanized by cardiac puncture. The lungs and spleen were flash-frozen and stored in −80°C freezer until further analysis. The serum was isolated from the blood obtained by the cardiac puncture and stored in −80°C freezer until IgE testing.

### 2.5. Bronchoalveolar Lavage (BAL)

A subset of mice (*n* = 42) distributed across each experimental group was chosen randomly for BAL instead of AHR measurement. The BAL fluid was centrifuged at 4°C at 1500 rpm for 5 minutes. Cell pellets were resuspended in 1 mL of PBS. Slides were prepared using a cytocentrifuge (Cytospin; Shandon GMI, Ramsey, MN) at 500 rpm for 5 minutes and then stained with Wright-Giemsa stain (Sigma-Aldrich, St. Louis, MO). Total 100 cells were counted for each sample from 10 randomly chosen viewing fields and total eosinophil, lymphocyte, macrophage, and neutrophil counts were quantified by a masked reader.

### 2.6. *β*_2_AR Gene Expression

Tissues for gene expression were derived from prenatal and postnatal PAH exposed mice across the four experimental groups. The prenatal specimens were derived from either the nonperfused lung that was ligated during the BAL (*n* = 13) or from lungs of mice that did not undergo AHR or BAL (*n* = 25) and were preserved in RNAlater (Ambion, Austin, TX) in the presence of Trizol reagent (Invitrogen, Carlsbad, CA). The postnatal specimens were derived from the nonperfused lung that was ligated during BAL (*n* = 22). To account for possible systemic changes in gene expression, corresponding spleens were collected from the same animals. Lungs and splenic DNA samples were pooled first by experimental group, and then the gene expression experiments in the lungs were repeated in individual samples. Precellys 24 homogenizer (Bertin Technologies, France) was used to homogenize mouse lung and spleen tissues at 5000 rpm for 15 sec and repeated twice. The RNA was extracted using SuperScript III Reverse Transcriptase (Invitrogen, Grand Island, NY) according to the manufacturer's (Invitrogen, Grand Island, NY) suggested protocol. Primers for real-time RT-PCR were designed by using Primer-BLAST program (http://www.ncbi.nlm.nih.gov/tools/primer-blast/). The sequences of *β*_2_AR forward and reverse primers are 5′-CTT GCT GGC ACC CAA CGG AA-3′ and 5′-ATG CCC ACA ACC CAC GCT TC-3′, respectively, with the amplicon size 78 bp. Mouse *β*_2_ microglobulin (*β*_2_m) was used as the housekeeping gene. The sequences of *β*_2_m forward and reverse primers are 5′-GCC TGT ATG CTA TCC AGA AAA CCC C-3′ and 5′-TGA GGC GGG TGG AAC TGT GT-3′, respectively, with the amplicon size 112 bp. Real-time RT-PCR was performed in a 7900HT Fast Real-Time PCR System (ABI, Foster City, CA) under standard mode. The RT-PCR was performed in triplicate by using three *μ*L 1 : 10 diluted cDNA in a total of 10 *μ*L reaction. The 2^−ΔΔCt^ method was used to calculate the relative expression level of *β*_2_AR transcripts as we performed previously [20].

### 2.7. *β*_2_AR DNA Methylation

Lungs and splenic DNA samples were pooled by experimental group. Genomic DNA was extracted from mouse lung and splenic tissue by DNeasy Blood & Tissue kit (Qiagen, Valencia, CA) in the presence of RNase A. EZ DNA Methylation kit (Zymo Research, Irvine, CA) was used to bisulfite convert 500 ng of genomic DNA in each reaction and then eluted with 40 *μ*L buffer. *β*_2_AR gene promoter flanking transcription start site was selected for the methylation analysis. Methprimer [21] was used to design primers for bisulfite PCR. The forward and reverse primers for *β*_2_AR bisulfite PCR are 5′-TTG GGT AAT TTT TTT AAA GTT TGG T-3′ and 5′-ACA TAA AAA TAA CCA TAC CCA CAA C-3′, respectively. The 390 bp amplicon (−40 tp +350) covers 32 CpG sites in the CpG island located in the region from +1 to +473.

PCR was performed by adding 2 *μ*L bisulfite modified DNA as template in a 25 *μ*L reaction. Platinum Taq DNA polymerase (Invitrogen, Carlsbad, CA) was added to the reaction according to manufacturer's instructions. The PCR together with the negative control were performed for 40 cycles with the annealing temperature set at 55°C. Gel-purified amplicons were then TA-cloned into pGEM T easy vector (Promega, Madison, WI). The plasmids in *E. coli* were directly amplified by TempliPhi DNA amplification kits (GE Healthcare, Indianapolis, IN) and submitted for sequencing (Macrogen USA, Rockville, MD). For each sample, a total of 12 clones were picked. The methylation status in each CpG site was analyzed by BiQ Analyzer [22].

### 2.8. Statistics

Differences between experimental groups (levels of pyrene collected on filters, Newtonian resistance) were assessed by Student's *t*-test and means and standard error values displayed. One-way ANOVA was used to analyze the AHR data. Generalized estimating equations (GEE) were used to evaluate R_*n*_ by experimental condition across all methacholine doses. Two-tailed *P* values <0.05 were considered statistically significant in all models.

## 3. Results

### 3.1. PAH Were Well-Aerosolized in Exposure Chamber

In order to verify proper aerosolization of the PAH in the exposure chamber, particulate fractions of the ambient PAH versus negative control aerosol were measured from the filters over 5-day periods and then averaged. Measured pyrene concentration in the PAH exposure group averaged higher than those in the negative control group (23.24 ± 3.05 versus 3.03 ± 0.90, *P* < 0.005) (Table 2). The experimental PAH concentration was on average 33-fold higher than the background PAH concentration in the animal housing facility (see Supplemental Table 1 available online at http://dx.doi.org/10.1155/2013/603581), documenting aerosolization of the PAH during experimental procedures. Time weighted concentrations for the experimentally exposed mice were approximately 74-fold higher than levels measured in the housing facility.

### 3.2. OVA-IgE and Airway Eosinophilia

In order to determine whether the mice were properly sensitized to OVA, anti-OVA IgE levels were measured from serum collected prior to sensitization and 10 days following the last i.p. dose. OVA sensitized mice had higher fold increases than those of the PBS sensitized mice (12.69 ± 2.71, 1.69 ± 0.29, mean ± SE for OVA, PBS control, resp., *P* = 0.003).

In the limited number of mice sampled (*n* = 19, selected randomly from 4 rounds of exposure), a greater percent of eosinophils were detected among the OVA sensitized mice when compared to the nonsensitized mice (10% + 1.14 versus 4% + 0.90, *P* = 0.003). There was no difference between cell numbers across all experimental groups of the postnatal exposed mice (data not shown).

### 3.3. Effects of Prenatal PAH Exposure in Wildtype Mice

**Figure 3:**
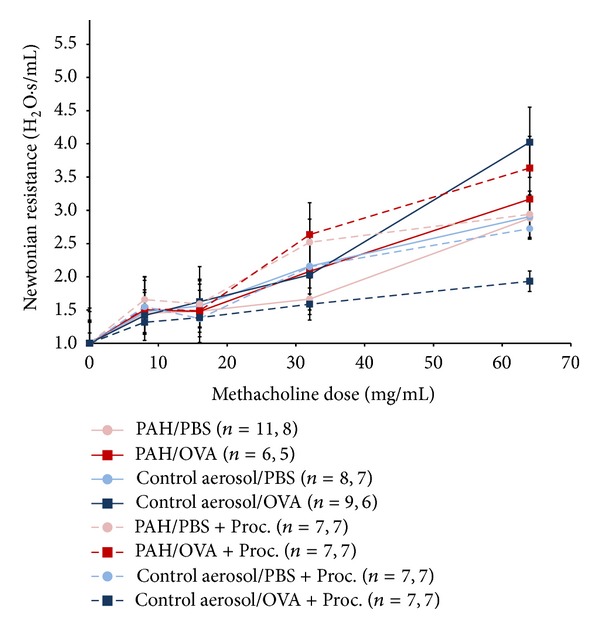
AHR following prenatal PAH exposure in the presence and absence of procaterol (Proc). Newtonian resistance (H_2_O/s/mL) was normalized to value measured following PBS aerosol exposure. Total sample size, followed by sample size for mice that completed highest dose, is listed in parentheses.

Airway reactivity data were analyzed by normalizing Newtonian resistance (H_2_O/s/mL) to the value measured following PBS aerosol exposure. Significant differences were not detected across any of the experimental groups (*P* = NS, ANOVA) (Figure 3). Experimental groups did not exhibit improvement in AHR following administration of procaterol (*P* = NS).

### 3.4. Effects of Early Postnatal PAH Exposure in Wildtype Mice

**Figure 4:**
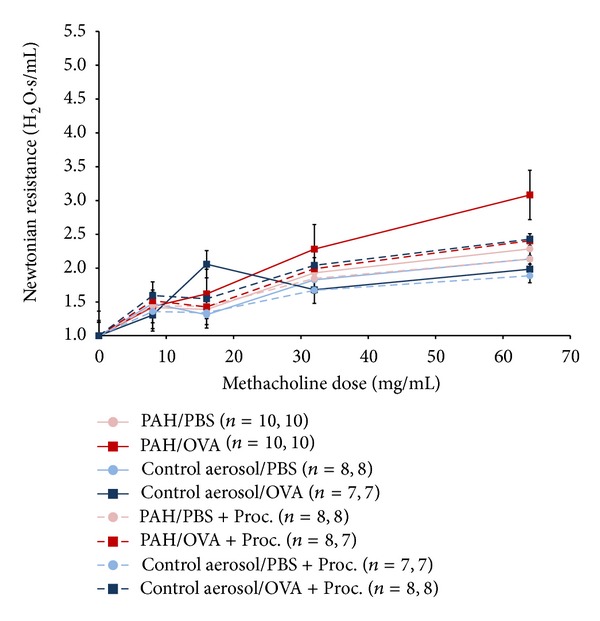
AHR following postnatal PAH exposure in the presence and absence of procaterol (Proc). Newtonian resistance (H_2_O/s/mL) was normalized to value measured following PBS aerosol exposure. Total sample size, followed by sample size for mice that completed highest dose, is listed in parentheses. The PAH/OVA mice showed borderline greater airway resistance when compared to the normal air/OVA group at the 64 mg/mL dose (*P* value = 0.055, two-tailed *t*-test). Clear improvement across experimental groups following administration of procaterol is not evident.

PAH/OVA mice exhibited borderline higher airway resistance when compared to the negative control aerosol/OVA exposure group (*P* = 0.055, two-tailed *t*-test) (Figure 4). This modest finding was confirmed in GEEs that showed a highly significant *β* value among the PAH/OVA that exceeded the value calculated from the other experimental groups (Supplemental Table 2). There was no significant resolution of AHR among mice following administration of procaterol, regardless of PAH exposure (*P* = NS).

### 3.5. Effects of Early Postnatal PAH Exposure Airway Results in *β*_2_AR^(−/−)^ Double Knockout (DKO) Mice

**Figure 5:**
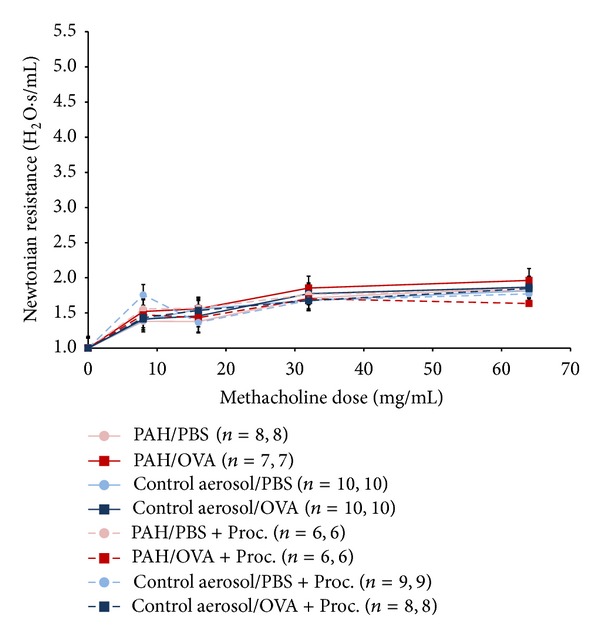
AHR following postnatal PAH exposure in the presence and absence of procaterol in *β*_2_AR^(−/−)^ DKO mice. There were no significant differences between the OVA sensitized/OVA challenged and the PBS sensitized/OVA challenged mice in either PAH or normal air exposure (*P* = NS). Clear improvement across experimental groups following administration of procaterol is not evident.

Significant differences in AHR were not detected across experimental groups in *β*_2_AR^(−/−)^ DKO mice (*P* = NS, ANOVA) (Figure 5). Further, significant improvements were not detected in any of the experiment groups following pretreatment with procaterol (*P* = NS).

### 3.6. *β*_2_AR Gene Expression and DNA Methylation

Finally, we sought to determine whether *β*_2_AR molecular function was impaired, even in the absence of detectable impairment of *β*_2_AR agonist physiological activity (i.e., improvement in AHR) with administration of procaterol. This question was examined through determination of whether aerosolized PAH altered *β*_2_AR gene expression and/or DNA methylation. We found that mice prenatally exposed to PAH (regardless of sensitization) exhibited borderline lower *β*_2_AR gene expression in the lung, but not spleen, when compared to the negative control aerosol mice (i.e., *P* = 0.054) (Table 3). Differences may have been more pronounced among the nonsensitized mice where the PAH/PBS mice showed lower *β*_2_AR gene expression than that of the negative control aerosol/PBS mice (*P* = 0.048) (Table 3). There were no other differences in gene expression between groups of prenatal exposed mice (*P* = NS). Also, no differences across experimental groups following postnatal exposure to PAH were detected (*P* = NS). There were no differential effects on DNA methylation in the lungs or spleen (unmethylated in all groups, data not shown).

## 4. Discussion

Using a novel chamber to determine the effects of a more physiological method of mouse exposure to ambient PAH, we found that prenatal exposure to PAH was associated with reduced *β*_2_AR gene expression in the mouse lung, and postnatal exposure to PAH was borderline associated with increased AHR. While these effects were modest or borderline, they may suggest a possible novel mechanism for loss of *β*_2_AR efficacy following ambient PAH exposure, a common urban pollutant. These results also extend our previous work in primary cells [15] and suggest the first *in vivo* demonstration of altered *β*_2_AR function in the lung following air pollution exposure. One major strength of this study includes the innovative delivery of ambient PAH exposure using individual PAH concentrations proportionate to exposure in the NYC setting [15] and not yet reported in other studies [18]. Other strengths include the assessment of the effects of PAH during the prenatal versus postnatal time periods, documentation of aerosolization through measurement of levels extracted from filters placed in the chamber and comparison with levels following nonexperimental (i.e., mouse housing) levels, and direct assessment of AHR.

The primary outcome of interest, namely, AHR, was only suggested following early (starting within 3 days of birth) postnatal PAH exposure. Clearly these results were of borderline statistical significance. In contrast, differences in AHR were not found following prenatal exposure, unlike previous human cohort studies [4, 6, 8]. We suspect that the effects of early postnatal PAH may mirror what we found in human cohorts where the combination of prenatal and early postnatal exposure to pyrene, the predominant local airborne PAH, was associated with childhood asthma and related symptoms [1]. Interesting, these results are the opposite of Singh and colleagues' findings following exposure of mice to secondhand smoke. In their model, prenatal, but not postnatal, secondhand smoke exposure increased methacholine-induced AHR in sensitized animals and upregulated allergic immune responses [23]. Fedulov and colleagues also showed that diesel exhaust particles during pregnancy induced greater AHR in the mice offspring [18].

It is not surprising that PAH-induced alterations in AHR may have been observed following postnatal exposure in mice, whereas PAH-induced asthma has been associated with prenatal exposure in some human cohorts [4]. In humans, lung alveolarization continues through age of 7 years, whereas, in the mouse, lung development starts prenatally and continues for several weeks after birth [18]. Hence, the period when rapidly proliferating cells may be most susceptible to toxic environmental exposures occurs during different times of exposure across species and may be later for mice. In addition, the mechanics of breathing differ between mouse and human. Small mammals, like mice, breathe faster [24]. Presumably higher ventilation rates may affect the dose of toxin inhaled.

Another possibility is that other toxicants or chemical compounds are responsible for the previously observed adverse associations following exposure to PAHs. These may include oxygenated PAH (that may increase with photooxidation processes when ambient ozone concentrations are high) and nitroPAHs that were not provided in the aerosol and exposure chamber. Reactive oxygen species may be present in ambient particulate matter or generated upon inhalation of PAHs in the lungs and can be converted to hydroxyl radicals that can induce mitochondrial damage and apoptosis [25, 26]. Also, several families of electrophilic metabolites (diol epoxides, radical cations, and reactive and redox active *o*-quinones) possess cytotoxic properties and have been implicated in PAH-induced carcinogenesis [24].

To ascertain whether the possible association with AHR was mediated by PAH-induced impairment of *β*_2_AR function, we conducted parallel experiments in *β*_2_AR KO mice that would not be expected to exhibit any *β*_2_AR function. With this model, we were unable to implicate the *β*_2_AR pathway as being causal. This result arguably was limited by the insufficient induction of AHR across the experimental groups, documented to occur in some *β*_2_AR KO mouse models [27, 28], which also made the determination of procaterol-induced effects difficult. Also, the intensity of AHR can vary by wildtype mouse strain, possibly attributable to strain-related differences in the expression of phospholipase C-*β*_1_ [27], airway inflammation [25], or calcium sensitivity [19].

Despite the absence of an effect of prenatal exposure to PAH on AHR and sensitivity to *β*_2_ adrenergic agonist drugs, differences across exposure groups in *β*_2_AR gene expression in the lung were evident. We had predicted that changes in gene expression could occur despite the absence of differences across groups in procaterol-induced resolution of AHR. This finding was more statistically sound when comparing the PAH versus negative control aerosol exposure groups among the nonsensitized mice, suggesting greater susceptibility to PAH exposure when allergic immune responses are not induced. Even though this finding occurred without downstream effects on airway bronchodilation as measured by AHR, the greater susceptibility in the absence of sensitization is consistent with our previous publication. In this work, exposure to pyrene was most associated with asthma and asthma-related symptoms among the nonatopic young children [1]. A similar pattern was observed in other human cohorts where exposure to secondhand smoke was associated with asthma symptoms in nonatopic children [29, 30]. Further, this finding is consistent with other examples in the literature that prenatal measures of several environmental exposures, including tobacco smoke, farming environment, and methyl-rich diet, affect asthma gene expression in the offspring [10, 31–33]. Epigenetic regulation of in *β*_2_AR gene expression in T helper 1 and 2 cells has been detected previously [33]. However, the differences in our experiments were not attributable to alterations in DNA methylation, suggesting that an alternative method influenced gene expression. These effects also appeared end organ specific, as they were not detected in the spleen.

We acknowledge several limitations. Differences across mouse strains were not tested, limiting the interpretation of the results from KO mice and comparisons across groups. The small sample sizes may have underpowered us to detect small differences across experimental groups, perhaps explaining the weak results following administration of procaterol. The experimental conditions may have been insufficient to induce more physiological or molecular effects. Finally, we were unable to compare AHR measures with gene expression measures because different mice necessarily were used to avoid the effects of AHR-related procedures on gene expression.

In conclusion, this is the first study to deliver PAH aerosols to mice in a relatively physiological manner and measure their effects on airway and molecular outcomes. This may be the first *in vivo* demonstration that a mixture of common traffic-related air pollutants may impair *β*_2_AR function on some level prenatally and postnatally, as the results begin to suggest that postnatal PAH exposure may induce AHR. Although the reduction in *β*_2_AR gene expression that was present following prenatal PAH exposure, particularly in the absence of OVA sensitization, was not associated with altered AHR or loss of reversibility after administration of a *β*_2_ agonist, hence, its clinical significance is unclear. If the molecular effects are confirmed and associated with other outcomes in future studies, they may help drive a novel paradigm for urban asthma morbidity.

## Supplementary Material

Table 1. Pyrene was well-aerosolized in exposure chamber The experimental PAH concentration was on average approximately 33 fold higher than the PAH concentration in the Medical Center animal housing facility. Using time weighted concentrations that considered weekly measures of PAH from 3 filters collected from the chamber each following 25 hours a week of experimental exposure (75 hours total) and extracted together versus one filter collected from the mouse housing facility collected after two weeks or 168 hours, the difference between experimental exposure and that measured during normal mouse housing exposure was 74 fold.
Table 2. Generalized estimating equation (GEE) evaluating Rn by experimental condition across all methacholine doses following postnatal exposure to PAH versus Negative Control Aerosol N depicts the number of mice. GEE model was used to evaluate Newtonian resistance by methacholine dose. All p values were < 0.005.

## Figures and Tables

**Table 1:** Components of PAH aerosol.

PAH	Proportion (%)	Mean (ng/m^3^)
Benzo[a]anthracene	3.99	0.27
Benzo[a]pyrene	5.13	0.42
Benzo[b]fluoranthene	7.62	0.59
Benzo[k]fluoranthene	1.79	0.15
Benzo[g,h,i]perylene	13.02	1.12
Chrysene	4.82	0.35
Dibenzo[a,h]anthracene	0.89	0.06
Indeno[c,d]pyrene	7.42	0.64
Pyrene	52.59	3.69

**Table 2:** Pyrene concentrations (ng/m^3^) in exposure chamber.

Exposure	*N*	Mean + SE	Range
Normal air	11	3.03 ± 0.90	0–9.00
PAH	11	23.24 ± 3.05	7.38–40.00

*N* represents the number of extractions of 3 filters, each one collecting pyrene levels over a 5-day period of aerosolization. The single level obtained from 3 consecutive filters was designed to capture the average level over a single exposure period. 11 filters were chosen from 13 rounds of mice representing both exposure groups evenly. Measured pyrene concentration in the PAH exposure group averaged higher than that in normal air exposure group (*P* < 0.005, two-tailed *t*-test).

**Table 3:** *β*_2_AR gene expression in lungs.

	Mean ± SE		*P* value
PAH versus normal air	0.65 ± 0.05 (*N* = 15)	0.91 ± 0.11 (*N* = 18)	0.054

PAH/PBS versus normal air/PBS	0.55 ± 0.06 (*N* = 7)	1.00 ± 0.18 (*N* = 9)	0.048

PAH/OVA versus normal air/OVA	0.75 ± 0.07 (*N* = 8)	0.83 ± 0.13 (*N* = 9)	0.620

*N* refers to the number of mice per group. Values were normalized by setting a mean expression level from the normal air/OVA group and comparing values with the other groups. PAH exposed mice exhibited lower *β*_2_AR gene expression than that of normal air exposed mice, regardless of sensitization (*P* = 0.054, two-tailed *t*-test). Differences in gene expression appeared more pronounced among the nonsensitized mice (*P* = 0.048, two-tailed *t*-test).
