# Supplementary material for: Prenatal and Postnatal Polycyclic Aromatic Hydrocarbon Exposure, Airway Hyperreactivity, and Beta-2 Adrenergic Receptor Function in Sensitized Mouse Offspring
Source: J Toxicol. 2013 Dec 12;2013:603581. doi: 10.1155/2013/603581 (PMC3876588; doi:10.1155/2013/603581)
Supplement: Supplementary file 1 — Table 1. Pyrene was well-aerosolized in exposure chamber The experimental PAH concentration was on average approximately 33 fold higher than the PAH concentration in the Medical Center animal housing facility. Using time weighted concentrations that considered weekly measures of PAH from 3 filters collected from the chamber each following 25 hours a week of experimental exposure (75 hours total) and extracted together versus one filter collected from the mouse housing facility collected after two weeks or 168 hours, the difference between experimental exposure and that measured during normal mouse housing exposure was 74 fold. Table 2. Generalized estimating equation (GEE) evaluating Rn by experimental condition across all methacholine doses following postnatal exposure to PAH versus Negative Control Aerosol N depicts the number of mice. GEE model was used to evaluate Newtonian resistance by methacholine dose. All p values were < 0.005. [file 603581.f1.pdf]

Table 1. Pyrene was well-aerosolized in exposure chamber

	Mean Pyrene Concentration (ng/m3)
Columbia University Morningside Animal Housing Facility	0.33
Columbia University Medical Center Animal Housing Facility	0.70
PAH Exposure Chamber	23.24
Negative Control Chamber	3.03

The experimental PAH concentration was on average approximately 33 fold higher than the PAH concentration in the Medical Center animal housing facility. Using time weighted concentrations that considered weekly measures of PAH from 3 filters collected from the chamber each following 25 hours a week of experimental exposure (75 hours total) and extracted together versus one filter collected from the mouse housing facility collected after two weeks or 168 hours, the difference between experimental exposure and that measured during normal mouse housing exposure was 74 fold.

Table 2. Generalized estimating equation (GEE) evaluating R_n by experimental condition across all methacholine doses following postnatal exposure to PAH versus Negative Control Aerosol

	N	Beta
PAH/OVA	10	0.032
PAH/PBS	10	0.019
Control aerosol/OVA	7	0.012
Control aerosol//PBS	8	0.016
PAH/OVA + Proc	8	0.020

PAH/PBS + Proc	8	0.016
Control aerosol//OVA + Proc	8	0.020
Control aerosol//PBS + Proc	7	0.013

N depicts the number of mice. GEE model was used to evaluate Newtonian resistance by methacholine dose. All p values were < 0.005.
